# 1-[(*E*)-4-(Phenyl­diazen­yl)phen­yl]-3-pyrroline-2,5-dione

**DOI:** 10.1107/S160053681103193X

**Published:** 2011-08-11

**Authors:** Elena Rusu, Sergiu Shova, Gheorghe Rusu

**Affiliations:** a Institute of Macromolecular Chemistry ‘Petru Poni’, Polymer Physics and Structure Department, 41A Grigore Ghica Voda Alley, Iasi-700487, Romania; b Institute of Applied Physics of the Academy of Science of Moldova, 5 Academiei Street, Chisinau MD-2028, Moldova

## Abstract

The title compound, C_16_H_11_N_3_O_2_, displays a *trans* configuration with respect to the azo group. The mol­ecule is non-planar; the maleimide ring forms a dihedral angle of 42.35 (4)° with the benzene ring bonded to its N atom and the mean plane of this benzene ring is rotated by 21.46 (8)° with respect to the azo group mean plane, which, in turn, forms a dihedral angle of 24.48 (7)° with the ‘terminal’ benzene ring. Mol­ecules in the crystal are π–π stacked along the [100] direction with a mean inter­planar distance of 3.857 (1) Å. In addition, C—H⋯O inter­actions link them into double layers parallel to the *ac* plane.

## Related literature

For studies of photo- and thermal isomerization of aromatic azo compounds, see: Serra & Terentjev (2008 ▶). For azocompounds based on maleimides, see: Mohammed & Mustapha (2010 ▶); Oishi *et al.* (2011 ▶). For the reactivity of the maleimide group, see: Knauf *et al.* (2004 ▶); Durmaz *et al.* (2006 ▶); Pounder *et al.* (2008 ▶).
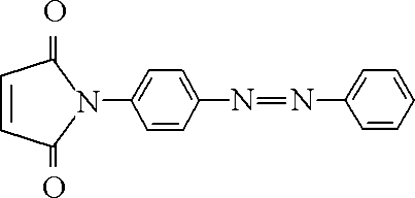


## Experimental

### 

#### Crystal data

C_16_H_11_N_3_O_2_
*M* *_r_* = 277.28
Triclinic, 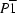

*a* = 3.8571 (2) Å
*b* = 10.9189 (7) Å
*c* = 15.784 (1) Å
α = 78.297 (5)°
β = 87.301 (5)°
γ = 88.809 (5)°
*V* = 650.18 (7) Å^3^
*Z* = 2
Mo *K*α radiation
μ = 0.10 mm^−1^
*T* = 200 K
0.20 × 0.15 × 0.15 mm

#### Data collection

Oxford Diffraction Xcalibur Eos diffractometer
Absorption correction: multi-scan (*CrysAlis PRO*; Oxford Diffraction, 2009 ▶) *T*_min_ = 0.981, *T*_max_ = 0.986
8689 measured reflections
2556 independent reflections
2189 reflections with *I* > 2σ(*I*)
*R*_int_ = 0.023

#### Refinement

*R*[*F* ^2^ > 2σ(*F* ^2^)] = 0.039
*wR*(*F* ^2^) = 0.107
*S* = 1.03
2556 reflections
190 parameters
H-atom parameters constrained
Δρ_max_ = 0.21 e Å^−3^
Δρ_min_ = −0.21 e Å^−3^

### 

Data collection: *CrysAlis PRO* (Oxford Diffraction, 2009 ▶); cell refinement: *CrysAlis PRO*; data reduction: *CrysAlis PRO*; program(s) used to solve structure: *SHELXS97* (Sheldrick, 2008 ▶); program(s) used to refine structure: *SHELXL97* (Sheldrick, 2008 ▶); molecular graphics: *ORTEP-3* (Farrugia, 1997 ▶); software used to prepare material for publication: *SHELXL97*.

## Supplementary Material

Crystal structure: contains datablock(s) I, global. DOI: 10.1107/S160053681103193X/ya2141sup1.cif

Structure factors: contains datablock(s) I. DOI: 10.1107/S160053681103193X/ya2141Isup2.hkl

Supplementary material file. DOI: 10.1107/S160053681103193X/ya2141Isup3.cml

Additional supplementary materials:  crystallographic information; 3D view; checkCIF report

## Figures and Tables

**Table 1:** Hydrogen-bond geometry (Å, °)

*D*—H⋯*A*	*D*—H	H⋯*A*	*D*⋯*A*	*D*—H⋯*A*
C2—H2⋯O2^i^	0.93	2.54	3.3841 (18)	152
C10—H10⋯O1^ii^	0.93	2.54	3.2464 (16)	133
C13—H13⋯O2^iii^	0.93	2.54	3.4248 (18)	160

Symmetry codes: (i) 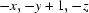; (ii) 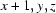; (iii) 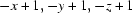.

**Fig. 1.**
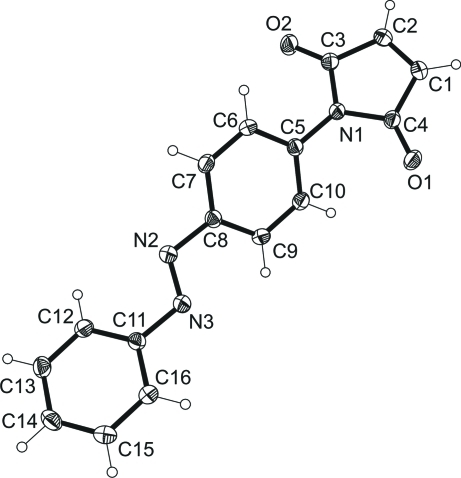
Molecular structure of the title compound; thermal ellipsoids are drawn at 40% probability level.

**Fig. 2.**
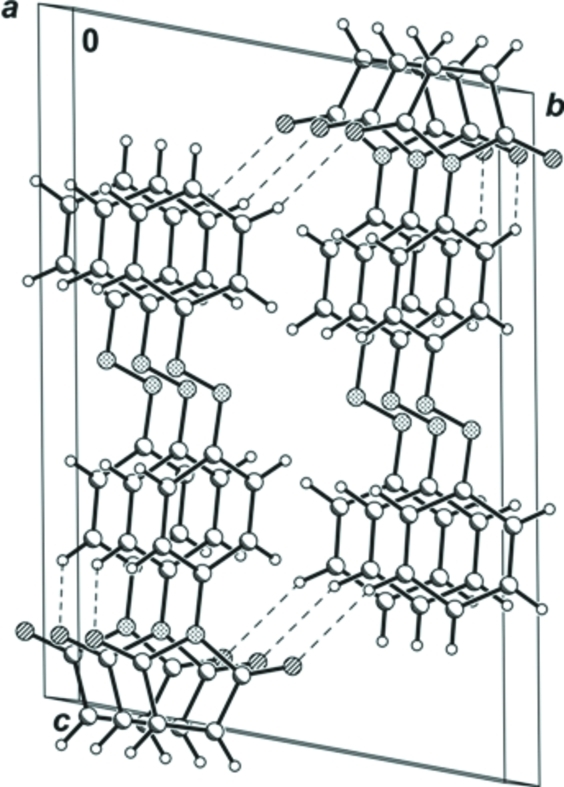
Crystal structure of the title compound viewed along the a axis; the C—H···O interactions are shown as dashed lines.

## supplementary crystallographic information

### Comment

As a part of our research study of the photosensitive compounds, we report the synthesis and crystal structure of the title compound, C_16_H_11_N_3_O_2_, which contains azobenzene and maleimide moieties. The importance of azobenzene chromophore in pure and applied chemistry as well as in nature is due to its photoswitchable properties and possibility to tune synthetically the wavelengths effecting the transformation of azocompounds (Serra & Terentjev, 2008; Mohammed & Mustapha, 2010; Oishi *et al.*, 2011). On the other hand, the presence of the electron-deficient double bond in the structure of maleimides determines the photoreactivity of these derivatives, *i.e.* photocycloaddition, polymerization, crosslinking, Diels-Alder, Michael-addition, click reactions (Knauf *et al.*, 2004; Durmaz *et al.*, 2006; Pounder *et al.*, 2008).

The molecular structure of the title compound is shown in Fig. 1. The configuration of this molecule in the crystal is *trans* with respect to azo bridge. The molecule is non-planar: the maleimide ring forms dihedral angle of 42.35 (4)° with the benzene ring C5—C10; the mean plane of this benzene ring is rotated by 12.46 (8)° with respect to the azo group mean plane, which, in its turn, forms the dihedral angle of 24.48 (7)° with the second benzene ring C11—C16.

The molecules form stacks along [100] due to π-π interactions. In addition, the weak C—H···O interactions contribute to self assembling of stacked molecules through the short contacts O2···C13^i^ = 3.425 (2) Å [symmetry code (i): 3 - *x*, 1 - *y*, 1 - *z*], O2···C2^ii^ = 3.384 (2) Å [symmetry code (ii): 2 - *x*, 1 - *y*, -*z*] and O1···C10^iii^ = 3.247 (2) Å [symmetry code (iii): *x* - 1, *y*, *z*] (Fig. 2). Thanks to the above mentioned interactions, molecules in crystal are linked into double layers parallel to the *ac*-plane.

### Experimental

Maleic anhydride (15 mmol, 1.47 g) was dissolved in dry acetone (15 ml) and a cold solution of 4-aminobenzoic acid (15 mmol, 2.96 g) in acetone (15 ml) was added under stirring to it at ice bath temperature. This mixture was stirred at room temperature for 3 h resulting in a white precipitate which was separated by filtration, washed several times with acetone and recrystallized from water to give maleamic acid of analytical purity. Then it was added to a solution of sodium acetate in acetic anhydride (30 ml, 0.025 *M*) in order to cyclize to the corresponding maleimide. The reaction was conducted under nitrogen at 80°C for 4 h. The mixture was poured into a saturated aqueous solution of NaHCO_3_ and then the precipitate was washed three times with water and dried at 50°C under vacuum. Pure (*E*)-4-(*N*-maleimido)azobenzene was obtained as a light orange crystalline solid after recrystalization from chloroform; yield 85%. The expected formula of C_16_H_11_N_3_O_2_ was confirmed; m.p.= 442 K; nitrogen analysis calculated for C_16_H_11_N_3_O_2_: N, 15.15%. Found: N, 15.10%, ^1^H NMR (DMSO-d6)δ (p.p.m.): 7.25 (s, 2H, CH═CH), 7.6–7.7 (m, 5H, Ar), 7.95 (d, 2H, Ar, adjacent to azo), 8.05 (d, 2H, Ar, adjacent to imide).

### Refinement

The H atoms were positioned geometrically and refined using a riding model approximation with C—H = 0.93 Å and *U*_iso_(H) = 1.2 times *U*_eq_(C).

### Crystal data

**Table:**

C_16_H_11_N_3_O_2_	*Z* = 2
*M**_r_* = 277.28	*F*(000) = 288
Triclinic, *P*1	*D*_x_ = 1.416 Mg m^−^^3^
Hall symbol: -P 1	Melting point: 442 K
*a* = 3.8571 (2) Å	Mo *K*α radiation, λ = 0.71073 Å
*b* = 10.9189 (7) Å	Cell parameters from 2982 reflections
*c* = 15.784 (1) Å	θ = 2.9–29.3°
α = 78.297 (5)°	µ = 0.10 mm^−^^1^
β = 87.301 (5)°	*T* = 200 K
γ = 88.809 (5)°	Prism, orange
*V* = 650.18 (7) Å^3^	0.20 × 0.15 × 0.15 mm

### Data collection

**Table:**

Oxford Diffraction Xcalibur Eos diffractometer	2556 independent reflections
Radiation source: Enhance (Mo) X-ray Source	2189 reflections with *I* > 2σ(*I*)
graphite	*R*_int_ = 0.023
Detector resolution: 16.1593 pixels mm^-1^	θ_max_ = 26.0°, θ_min_ = 2.9°
ω scans	*h* = −4→4
Absorption correction: multi-scan (*CrysAlis PRO*; Oxford Diffraction, 2009)	*k* = −13→13
*T*_min_ = 0.981, *T*_max_ = 0.986	*l* = −19→19
8689 measured reflections	

### Refinement

**Table:**

Refinement on *F*^2^	Primary atom site location: structure-invariant direct methods
Least-squares matrix: full	Secondary atom site location: difference Fourier map
*R*[*F*^2^ > 2σ(*F*^2^)] = 0.039	Hydrogen site location: inferred from neighbouring sites
*wR*(*F*^2^) = 0.107	H-atom parameters constrained
*S* = 1.03	*w* = 1/[σ^2^(*F*_o_^2^) + (0.0625*P*)^2^ + 0.1268*P*] where *P* = (*F*_o_^2^ + 2*F*_c_^2^)/3
2556 reflections	(Δ/σ)_max_ < 0.001
190 parameters	Δρ_max_ = 0.21 e Å^−^^3^
0 restraints	Δρ_min_ = −0.21 e Å^−^^3^

### Special details

**Table:**

Geometry. All e.s.d.'s (except the e.s.d. in the dihedral angle between two l.s. planes) are estimated using the full covariance matrix. The cell e.s.d.'s are taken into account individually in the estimation of e.s.d.'s in distances, angles and torsion angles; correlations between e.s.d.'s in cell parameters are only used when they are defined by crystal symmetry. An approximate (isotropic) treatment of cell e.s.d.'s is used for estimating e.s.d.'s involving l.s. planes.
Refinement. Refinement of *F*^2^ against ALL reflections. The weighted *R*-factor *wR* and goodness of fit *S* are based on *F*^2^, conventional *R*-factors *R* are based on *F*, with *F* set to zero for negative *F*^2^. The threshold expression of *F*^2^ > σ(*F*^2^) is used only for calculating *R*-factors(gt) *etc*. and is not relevant to the choice of reflections for refinement. *R*-factors based on *F*^2^ are statistically about twice as large as those based on *F*, and *R*- factors based on ALL data will be even larger.

### Fractional atomic coordinates and isotropic or equivalent isotropic displacement parameters (Å^2^)

**Table:**

	*x*	*y*	*z*	*U*_iso_*/*U*_eq_	
N2	0.2043 (3)	0.69925 (10)	0.48218 (7)	0.0266 (3)	
N1	−0.0181 (3)	0.74413 (10)	0.12701 (7)	0.0268 (3)	
O1	−0.2299 (3)	0.94847 (8)	0.09406 (6)	0.0328 (3)	
C6	−0.0493 (3)	0.62500 (12)	0.27614 (8)	0.0262 (3)	
H6	−0.1515	0.5594	0.2574	0.031*	
N3	0.2360 (3)	0.80091 (10)	0.50624 (7)	0.0273 (3)	
C9	0.2492 (3)	0.82310 (12)	0.33156 (9)	0.0256 (3)	
H9	0.3477	0.8894	0.3504	0.031*	
C10	0.1985 (3)	0.83144 (12)	0.24472 (8)	0.0259 (3)	
H10	0.2662	0.9027	0.2048	0.031*	
C7	0.0096 (3)	0.61594 (12)	0.36306 (9)	0.0269 (3)	
H7	−0.0471	0.5430	0.4026	0.032*	
C5	0.0458 (3)	0.73313 (12)	0.21706 (8)	0.0240 (3)	
C11	0.3041 (3)	0.78708 (12)	0.59567 (8)	0.0251 (3)	
O2	0.1582 (3)	0.54706 (9)	0.10682 (7)	0.0440 (3)	
C16	0.2175 (4)	0.88877 (13)	0.63313 (9)	0.0297 (3)	
H16	0.1262	0.9613	0.5998	0.036*	
C12	0.4561 (4)	0.68014 (13)	0.64430 (9)	0.0287 (3)	
H12	0.5199	0.6130	0.6186	0.034*	
C8	0.1530 (3)	0.71524 (12)	0.39132 (8)	0.0243 (3)	
C13	0.5111 (4)	0.67469 (14)	0.73067 (9)	0.0334 (3)	
H13	0.6139	0.6040	0.7634	0.040*	
C4	−0.1609 (3)	0.84996 (12)	0.07348 (8)	0.0260 (3)	
C3	0.0284 (4)	0.64799 (13)	0.08024 (9)	0.0306 (3)	
C1	−0.2072 (4)	0.81469 (13)	−0.01158 (9)	0.0318 (3)	
H1	−0.2962	0.8667	−0.0600	0.038*	
C15	0.2678 (4)	0.88172 (14)	0.72036 (9)	0.0334 (3)	
H15	0.2043	0.9486	0.7463	0.040*	
C2	−0.1022 (4)	0.69835 (13)	−0.00739 (9)	0.0339 (3)	
H2	−0.1085	0.6546	−0.0520	0.041*	
C14	0.4132 (4)	0.77460 (14)	0.76871 (9)	0.0345 (3)	
H14	0.4454	0.7696	0.8273	0.041*	

### Atomic displacement parameters (Å^2^)

**Table:**

	*U*^11^	*U*^22^	*U*^33^	*U*^12^	*U*^13^	*U*^23^
N2	0.0303 (6)	0.0253 (6)	0.0246 (6)	0.0005 (4)	−0.0026 (5)	−0.0062 (5)
N1	0.0366 (6)	0.0216 (6)	0.0225 (6)	0.0040 (5)	−0.0046 (5)	−0.0052 (4)
O1	0.0456 (6)	0.0215 (5)	0.0303 (5)	0.0056 (4)	−0.0011 (4)	−0.0038 (4)
C6	0.0319 (7)	0.0201 (6)	0.0276 (7)	−0.0005 (5)	−0.0036 (5)	−0.0070 (5)
N3	0.0327 (6)	0.0253 (6)	0.0242 (6)	0.0025 (5)	−0.0027 (5)	−0.0056 (5)
C9	0.0281 (7)	0.0222 (7)	0.0277 (7)	−0.0009 (5)	−0.0005 (5)	−0.0079 (5)
C10	0.0292 (7)	0.0218 (7)	0.0257 (7)	0.0001 (5)	0.0016 (5)	−0.0031 (5)
C7	0.0330 (7)	0.0201 (6)	0.0263 (7)	0.0012 (5)	−0.0006 (5)	−0.0019 (5)
C5	0.0272 (7)	0.0231 (7)	0.0218 (6)	0.0057 (5)	−0.0017 (5)	−0.0055 (5)
C11	0.0263 (6)	0.0254 (7)	0.0238 (7)	−0.0025 (5)	−0.0023 (5)	−0.0049 (5)
O2	0.0742 (8)	0.0275 (6)	0.0324 (6)	0.0184 (5)	−0.0128 (5)	−0.0105 (4)
C16	0.0344 (7)	0.0245 (7)	0.0304 (7)	0.0009 (5)	−0.0034 (6)	−0.0060 (6)
C12	0.0317 (7)	0.0238 (7)	0.0315 (7)	0.0005 (5)	−0.0048 (6)	−0.0065 (6)
C8	0.0250 (6)	0.0245 (7)	0.0232 (7)	0.0033 (5)	−0.0017 (5)	−0.0048 (5)
C13	0.0360 (8)	0.0311 (8)	0.0313 (8)	−0.0013 (6)	−0.0109 (6)	0.0002 (6)
C4	0.0288 (7)	0.0229 (7)	0.0250 (7)	0.0000 (5)	−0.0011 (5)	−0.0021 (5)
C3	0.0424 (8)	0.0247 (7)	0.0258 (7)	0.0038 (6)	−0.0046 (6)	−0.0076 (5)
C1	0.0404 (8)	0.0289 (7)	0.0254 (7)	−0.0006 (6)	−0.0083 (6)	−0.0023 (6)
C15	0.0366 (8)	0.0341 (8)	0.0328 (8)	−0.0014 (6)	−0.0009 (6)	−0.0148 (6)
C2	0.0484 (9)	0.0303 (8)	0.0248 (7)	0.0001 (6)	−0.0070 (6)	−0.0085 (6)
C14	0.0371 (8)	0.0430 (9)	0.0246 (7)	−0.0071 (6)	−0.0062 (6)	−0.0080 (6)

### Geometric parameters (Å, °)

**Table:**

N2—N3	1.2539 (15)	C11—C16	1.3879 (19)
N2—C8	1.4320 (16)	C11—C12	1.3952 (19)
N1—C3	1.4046 (17)	O2—C3	1.2044 (17)
N1—C4	1.4049 (17)	C16—C15	1.3860 (19)
N1—C5	1.4346 (16)	C16—H16	0.9300
O1—C4	1.2062 (16)	C12—C13	1.3786 (19)
C6—C7	1.3845 (18)	C12—H12	0.9300
C6—C5	1.3918 (18)	C13—C14	1.386 (2)
C6—H6	0.9300	C13—H13	0.9300
N3—C11	1.4252 (16)	C4—C1	1.4893 (19)
C9—C10	1.3781 (18)	C3—C2	1.4882 (19)
C9—C8	1.3960 (18)	C1—C2	1.315 (2)
C9—H9	0.9300	C1—H1	0.9300
C10—C5	1.3893 (18)	C15—C14	1.384 (2)
C10—H10	0.9300	C15—H15	0.9300
C7—C8	1.3874 (18)	C2—H2	0.9300
C7—H7	0.9300	C14—H14	0.9300
			
N3—N2—C8	112.96 (11)	C13—C12—H12	120.2
C3—N1—C4	109.32 (10)	C11—C12—H12	120.2
C3—N1—C5	125.35 (11)	C7—C8—C9	119.91 (12)
C4—N1—C5	125.18 (11)	C7—C8—N2	117.05 (11)
C7—C6—C5	119.44 (12)	C9—C8—N2	123.01 (11)
C7—C6—H6	120.3	C12—C13—C14	119.98 (13)
C5—C6—H6	120.3	C12—C13—H13	120.0
N2—N3—C11	113.94 (11)	C14—C13—H13	120.0
C10—C9—C8	120.04 (12)	O1—C4—N1	125.76 (12)
C10—C9—H9	120.0	O1—C4—C1	128.12 (12)
C8—C9—H9	120.0	N1—C4—C1	106.12 (11)
C9—C10—C5	119.80 (12)	O2—C3—N1	125.56 (12)
C9—C10—H10	120.1	O2—C3—C2	128.17 (13)
C5—C10—H10	120.1	N1—C3—C2	106.24 (11)
C6—C7—C8	120.25 (12)	C2—C1—C4	109.21 (12)
C6—C7—H7	119.9	C2—C1—H1	125.4
C8—C7—H7	119.9	C4—C1—H1	125.4
C10—C5—C6	120.52 (12)	C14—C15—C16	119.64 (13)
C10—C5—N1	119.59 (11)	C14—C15—H15	120.2
C6—C5—N1	119.88 (11)	C16—C15—H15	120.2
C16—C11—C12	120.39 (12)	C1—C2—C3	109.09 (12)
C16—C11—N3	116.13 (11)	C1—C2—H2	125.5
C12—C11—N3	123.46 (12)	C3—C2—H2	125.5
C15—C16—C11	119.71 (13)	C15—C14—C13	120.69 (13)
C15—C16—H16	120.1	C15—C14—H14	119.7
C11—C16—H16	120.1	C13—C14—H14	119.7
C13—C12—C11	119.52 (13)		
			
C8—N2—N3—C11	−177.11 (10)	C10—C9—C8—N2	178.76 (11)
C8—C9—C10—C5	1.12 (19)	N3—N2—C8—C7	−160.38 (12)
C5—C6—C7—C8	1.8 (2)	N3—N2—C8—C9	21.50 (18)
C9—C10—C5—C6	−1.5 (2)	C11—C12—C13—C14	0.5 (2)
C9—C10—C5—N1	177.72 (11)	C3—N1—C4—O1	−179.48 (13)
C7—C6—C5—C10	0.0 (2)	C5—N1—C4—O1	4.7 (2)
C7—C6—C5—N1	−179.20 (11)	C3—N1—C4—C1	0.59 (15)
C3—N1—C5—C10	140.32 (14)	C5—N1—C4—C1	−175.19 (12)
C4—N1—C5—C10	−44.57 (18)	C4—N1—C3—O2	176.90 (15)
C3—N1—C5—C6	−40.51 (19)	C5—N1—C3—O2	−7.3 (2)
C4—N1—C5—C6	134.60 (14)	C4—N1—C3—C2	−1.19 (16)
N2—N3—C11—C16	−157.20 (12)	C5—N1—C3—C2	174.57 (12)
N2—N3—C11—C12	24.26 (18)	O1—C4—C1—C2	−179.59 (14)
C12—C11—C16—C15	−3.0 (2)	N1—C4—C1—C2	0.34 (16)
N3—C11—C16—C15	178.44 (12)	C11—C16—C15—C14	1.8 (2)
C16—C11—C12—C13	1.8 (2)	C4—C1—C2—C3	−1.07 (17)
N3—C11—C12—C13	−179.72 (12)	O2—C3—C2—C1	−176.60 (16)
C6—C7—C8—C9	−2.2 (2)	N1—C3—C2—C1	1.43 (17)
C6—C7—C8—N2	179.63 (11)	C16—C15—C14—C13	0.5 (2)
C10—C9—C8—C7	0.7 (2)	C12—C13—C14—C15	−1.6 (2)

### Hydrogen-bond geometry (Å, °)

**Table:**

*D*—H···*A*	*D*—H	H···*A*	*D*···*A*	*D*—H···*A*
C2—H2···O2^i^	0.93	2.54	3.3841 (18)	152
C10—H10···O1^ii^	0.93	2.54	3.2464 (16)	133
C13—H13···O2^iii^	0.93	2.54	3.4248 (18)	160

Symmetry codes: (i) −*x*, −*y*+1, −*z*; (ii) *x*+1, *y*, *z*; (iii) −*x*+1, −*y*+1, −*z*+1.
